# COVID-19 Vaccination: Status and Willingness to Be Vaccinated among Employees in Health and Welfare Care in Germany

**DOI:** 10.3390/ijerph18136688

**Published:** 2021-06-22

**Authors:** Agnessa Kozak, Albert Nienhaus

**Affiliations:** 1 Competence Center for Epidemiology and Health Services Research for Healthcare Professionals (CVcare), Institute for Health Services Research in Dermatology and Nursing (IVDP), University Medical Center Hamburg-Eppendorf (UKE), 20246 Hamburg, Germany; a.nienhaus@uke.de; 2 Department for Occupational Medicine, Hazardous Substances and Health Sciences (AGG), German Social Accident Insurance for the Health and Welfare Services (BGW), 22089 Hamburg, Germany

**Keywords:** COVID-19 vaccination, vaccination willingness, health workers, vaccine preference, online survey, occupational health

## Abstract

Healthcare workers are at particular risk due to their occupational exposure to SARS-CoV-2. Therefore, they belong to the top priority group for vaccination. However, earlier studies show that nursing staff in particular are hesitant to be inoculated. This study presents the current picture with regard to vaccination status, willingness, vaccine preference, and reasons for or against a COVID-19 vaccination among health and welfare workers. An online survey was conducted between 4 March and 10 April 2021 among professional associations and providers of health and social services. Data sets of *n* = 3401 participants were analyzed. Of these, 62% stated that they had already been vaccinated at least once. A further 22% wanted to be vaccinated, while 6.6% were still hesitant and 9% refused to be vaccinated. Preference was given to predominantly mRNA-based vaccines. Altogether, there was a high vaccination rate and a great willingness to be vaccinated (>80%) across all professional groups and fields of work. Among nursing staff, the total figure was 83.5%. The percentage was highest in geriatric care at 87.5%. Contrary to findings of earlier surveys, vaccination willingness has risen in all professional groups during the course of the vaccination campaign in Germany.

## 1. Introduction

After the WHO declared COVID-19 a pandemic in March 2020, research into vaccines against SARS-CoV-2 intensified [1]. Within a year, four vaccines with two different mechanisms of action were approved by the European Medicines Agency (EMA; as of 11 March 2021) [2]. There was a great deal of uncertainty among the population because the new vaccines were approved at record speed. On social networks in particular, fears and misinformation were spread, purporting that mRNA could change the human genetic code, reduce fertility, or cause cancer [3]. Furthermore, in the course of the vaccination drive, a conspicuous increase in a specific type of rare cerebral venous thrombosis (CVT) was seen in conjunction with thrombocytopenia and bleeding following inoculation with the Vaxzervria vaccine [4]. In Germany, this prompted the relevant body (Paul-Ehrlich-Institut) to halt vaccinations on 15 March 2021 [5]. After a review of the CVT cases, it was recommended in Germany that the vaccine be limited to the over-60 population and that people under 60 who had already received one dose should be given an mRNA-based vaccine [6].

Healthcare workers (HCWs) are on the front lines of the fight against the pandemic. Especially at the beginning of the pandemic, many HCWs became infected with SARS-CoV-2. About 14% of COVID-19 cases among HCWs were reported to the World Health Organization (WHO) in 2020. In some countries, the proportion was as high as 35% [7]. Despite substantial variation among countries and limitation of the data, the median death rate among HCW was estimated to be 0.05 cases per 100,000 population of the country. With 0.35 cases per 100,000 population, Italy has shown the highest mortality rate among HCWs in Europe [8]. According to the National Institute for Accident Insurance in Italy (as of 30 September 2020), 70% of all reported cases of COVID-19 concerned the “Health and Social Assistance” sector. The occupational groups most affected were health technicians (39%), followed by social health workers (20%) and physicians (10%). Correspondingly high mortality rates were documented for these occupations [9]. A recent meta-analysis showed that the prevalence of hospitalization and mortality in HCWs was 15% and 1.5%, respectively. Wearing personal protective equipment (PPE) and training in PPE use were found to be protective, whereas lack of N95/FFP2 masks, reused PPE, and insufficient hand hygiene were risk factors for SARS-Cov-2 infection [10]. In response to this devastating situation, WHO is calling for better protection of HCWs worldwide, ensuring that all vulnerable HCWs are vaccinated and have priority access to newly licensed and available vaccines [7].

The control of a pandemic depends on several factors. In addition to containment measures, contact tracing, and the effectiveness of the vaccines used, vaccination readiness in the population and especially in high-risk occupations is also crucial. In late 2020 and early 2021, HCWs from a range of fields in Germany were asked about their vaccination willingness. Initially, vaccination willingness was rather low (between 57% and 64%), but it increased to 76% as the vaccination campaign progressed [11,12,13,14]. Acceptance was markedly higher among physicians than among nursing staff (81% vs. 71%). On both survey dates, vaccination willingness was higher among men than women overall (73% and 81% vs. 54% and 73%, respectively) [12]. Nursing staff in particular—predominantly women—stated that they had serious concerns about side effects and long-term consequences [13,15,16]. A scoping review of vaccination willingness among HCWs shows that an average of 22.5% refuse to be vaccinated against COVID-19 or remain hesitant. Concerns about the safety, efficacy, and side effects of the coronavirus vaccines were the most common reasons for hesitating or refusing immunization [17]. In addition, individuals who reported having been vaccinated against influenza in the past were more likely to be vaccinated against COVID-19 [17,18,19].

When the survey was conducted, the first cases of rare side effects following vaccination with Vaxzervria had just become known. The resulting suspension of vaccinations and the revised recommendations that followed may also have impacted the willingness of workers in nursing and social professions to be inoculated. Other aspects such as the innovative technology used in the mRNA-based vaccines, the EMA’s rapid approval process, and the relatively short post-vaccination observation periods from the phase III trials could cause individuals to hesitate or refuse inoculation, especially in connection with the safety of the vaccines being used.

The objective of this survey was to obtain an up-to-date picture of vaccination rates and vaccination willingness among health and welfare workers by demographic factors, occupational group, field of work, place of residence (by state), influenza vaccination status, and contact to COVID-19 patients. In addition, reasons for and against COVID-19 vaccination, perceived willingness to vaccinate in the workplace, attitudes toward mandatory vaccination, and information acquisition about COVID-19 vaccines were identified to better address needs, preferences, and concerns during the vaccination campaign in Germany. The survey was extended to other professional groups identified as having high vaccination priority (e.g., staff working in welfare professions).

## 2. Materials and Methods

### 2.1. Sample

An anonymous online survey of health and welfare workers was conducted between 4 March and 10 April 2021. Members of these professions are primarily considered to be a priority group for immunization against COVID-19. The link to the survey was published on the website of the German Social Accident Insurance for the Health and Welfare Services (BGW). A request to participate was also issued via the BGW’s monthly newsletter. Furthermore, the survey link was forwarded to various professional associations and providers of health and social services. A total of 3429 people completed the online survey. Respondents who did not provide complete socio-demographic details or were retired (*n* = 28) were excluded from the subsequent analyses. This meant that 3401 data sets were available to us. A response rate could not be calculated because it was not possible to establish a clearly defined number of potential respondents in advance.

### 2.2. Questionnaire

A standardized questionnaire with 18 questions was self-developed and pre-tested on 12 individuals to assess the comprehensibility, applicability, and appropriateness of the questions as well as time required to complete the questionnaire. The aspects examined in the questionnaire were guided by questions from the field and previously conducted studies on COVID-19 vaccine readiness [13,15,19]. The questionnaire was structured into the following subsections:

First, participants were asked about vaccination status, willingness to be vaccinated, and preference of vaccines licensed in the EU at the time of the survey. They were asked to indicate whether they had already been vaccinated at least once and, if so, which vaccines they were administered. Apart from the question concerning vaccination status, all questions were optional. Individuals who were not yet vaccinated were asked about their willingness to be vaccinated (“yes, immediately”, “rather yes, but wait and see”, “undecided”, “rather no”, “no in any case”). Finally, participants were asked, if given a choice, which vaccine they would choose (“mRNA-based,” “vector-based”, “doesn’t matter, as long as I get vaccinated,” or “with none of the mentioned vaccines”).

**Figure 1:**
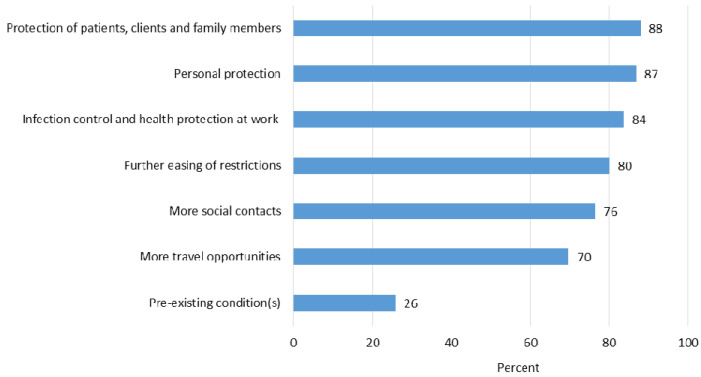
Reasons for COVID-19 vaccination (strongly agree/agree; percentage relative to the total sample).

**Figure 2:**
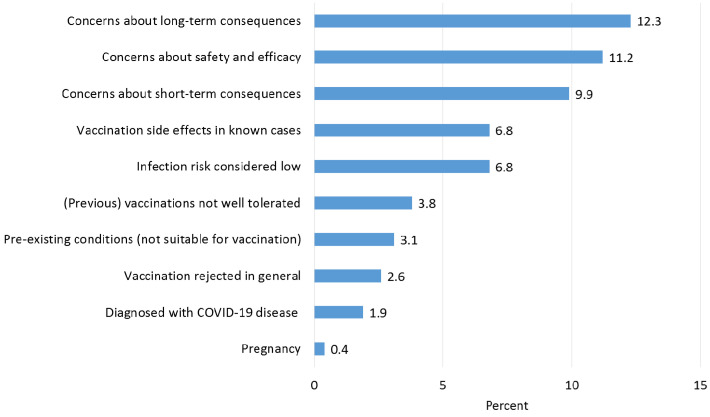
Reasons for hesitating or refusing COVID-19 vaccination (strongly agree/agree; percentage relative to the total sample).

In the second part, we asked about motivational reasons (7 statements) and reasons for refusal or hesitation (10 statements) to receive COVID-19 vaccination (see also Figure 1 and Figure 2). The questions could be answered on a 5-point Likert scale from 1 = “strongly agree” to 5 = “strongly disagree”.

In the third part, participants were asked to rate vaccination willingness at their facility (“high”, “moderate”, “low”, “don’t know”). If they rated vaccination readiness as moderate or low, we asked them to indicate what they thought might promote employee vaccination readiness. Six answer options were given, which could be selected in case of agreement: (1) “providing detailed information on the effectiveness and safety of the new COVID-19 vaccines”, (2) “transparent reporting on possible side effects and long-term consequences”, (3) “prolonged observation time of the vaccination process to better evaluate side effects and long-term consequences”, (4) “evidence of fewer COVID-19 cases in previously vaccinated individuals”, (5) “financial incentive”, or (6) “none of the above”. Other specific reasons and suggested measures could be entered as free text. These were grouped together in superordinate categories and evaluated based on the frequency with which they were mentioned.

According to the Robert Koch Institute, a public health institute in Germany, there are no plans for mandatory vaccination against COVID-19 [20]. We therefore asked whether the participants considered this approach to be reasonable. The response categories ranged from “yes in any case” to “no in no case”.

Personal details included gender, age, field of work, professional group, and federal state (place of residence). Respondents were also asked whether they had cared for or treated COVID-19 patients/clients in the past four weeks (response categories: “never”, “rarely”, “often”, “always”) and whether they had been vaccinated against seasonal influenza in the past five years (response categories: “every year”, “once or irregularly”, “none”).

The respondents were informed that data would be collected and analyzed anonymously and that data would only be processed with their consent. Answering the questions was voluntary and participation could be terminated at any time. Respondents were informed that the findings would be published in aggregated form. Tivian XI GmbH EFS online survey software was used for this questionnaire (Tivian XI GmbH, Köln, Germany) (https://www.unipark.com/umfragesoftware/, accessed on 21 June 2021).

### 2.3. Ethical Consideration

In accordance with the Professional Code for Physicians in Hamburg (Art. 15, 1., Status of 10 March 2014) and the Chamber Legislation for Medical Professions in the Federal State of Hamburg (HmbKGH) it is only necessary to obtain advice on questions of professional ethics and professional conduct from an Ethics Committee if data that can be traced to a particular individual is being used in a research project. All data in this trial were collected, analyzed, and disclosed anonymously, following the terms of the German Federal Data Protection Act (BDSG) and HmbDSG.

### 2.4. Statistics

The data underwent descriptive analysis and frequencies were presented separately based on vaccination status and/or willingness. Group differences were examined using a chi-squared test; the significance level was set at *p* < 0.05. To explore the potential factors for vaccination hesitancy or refusal, we used a stepwise backward logistic regression analysis. All variables that had a *p*-value of <0.25 in the bivariate analysis were included and then the least significant variables were removed stepwise. However, gender and age were included in the analysis regardless of significance level. The dependent variable was dichotomized as follows: “vaccinated or willing to be vaccinated” and “hesitant, undecided, and refusing”. SPSS version 27 was used to evaluate the data.

## 3. Results

### 3.1. Description of the Study Population

The vast majority of the respondents were women (70%) and 86% were over the age of 35. Of the respondents, 30% worked in geriatric care, 10% in nursing, 13% in disabled care, 11.3% in social work, and 35% in various other sectors (e.g., pharmacies, dentistry, therapeutic care, childcare, or hairdressing). The majority of the respondents worked in nursing (27%) and administration (29%). Medical staff accounted for a comparatively small proportion of 4.4%. At 86%, the majority of the respondents came from the former West German states (Table 1).

### 3.2. Vaccination Status, Vaccination Willingness, and Vaccine Preference

On the time of the survey, 62% of the participants had received at least one dose of a vaccine. BioNTech/Pfizer (54%) and AstraZeneca (41%) were the most frequently administered vaccines. A further 22% wanted to be vaccinated as soon as possible, 7% were hesitating or undecided, and 9% were inclined to refuse or had completely ruled out having a COVID-19 vaccination.

Those who were not vaccinated at the time of the study preferred mRNA-based vaccines (55%); few preferred vector-based vaccines (2%). Meanwhile, 23% had no preference (they just wanted to be vaccinated) and a further 21% did not want to be inoculated with any of the vaccines available at the time of the survey. The suspension of Vaxzervria (AstraZeneca) vaccinations on 15 March 2021 did not have a significant impact on the participants’ vaccination willingness, but it did affect their choice of vaccine. Prior to the suspension, 48% of respondents wanted to receive an mRNA vaccine. Afterwards, the figure stood at 57%. While 4% preferred viral vector vaccines beforehand, only 1.2% did afterwards (no Table).

The proportion of vaccinated and accepting individuals among women and men (including “diverse”) was roughly the same (84% and 85%, respectively). Of the under-35 group, 25% were hesitant or refused a COVID-19 vaccination, contrasting with the 35 to 55 age group (16%) and the over-55 group (11%). The highest percentage of vaccinated individuals was seen among nursing staff at 75%. A further 9% wanted to be vaccinated as soon as possible and 7% were hesitant or undecided. Just 10% of the nursing staff did not want to be inoculated. Vaccination willingness was very high overall among all other professions, even though groups such as educational staff and body-related service staff had the lowest vaccination rates at the time of the survey (Table 2).

An examination based on fields of work showed that geriatric caregivers had the highest vaccination rate at 81%, with a further 7% wanting to be inoculated as soon as possible. In disabled care and social work, 60% and 40%, respectively, had been vaccinated at the time of the study. However, taken together with the number who accepted inoculation, these groups had a similarly high vaccination willingness to workers in geriatric care and nursing. There were also regional differences in vaccination willingness. While 86% in the western federal states were already vaccinated or were willing to be inoculated straight away, the figure was 78% in the eastern states. Persons who either regularly or occasionally received vaccinations against the seasonal flu were significantly more likely to state that they would also be inoculated against COVID-19. However, of the group that had not received flu vaccinations in the past five years, 55% had been vaccinated against COVID-19 and a further 22% wanted to be inoculated (Table 2).

A total of 185 individuals stated that they had been in contact with COVID-19 patients or clients either often or occasionally in the past four weeks. Of these, 68% had already been vaccinated, a further 8.6% wanted to be vaccinated, 6.5% were hesitant, and 17% refused to be inoculated (Table 2).

Compared with participants older than 55 years, the odds ratios (OR) for hesitating/rejecting COVID-19 vaccine were 1.9 (95% CI: 1.40–2.59) in participants younger than 35 years and 1.3 (95% CI: 1.04–1.69) in participants aged 35–55 years (Table 3). Administrative (OR 0.6, 95% CI: 0.47–0.96) and educational/support staff (OR 0.5, 95% CI: 0.34–0.78) hesitated less often than participants in other occupational groups. Participants in geriatric care (OR 0.4, 95% CI: 0.30–0.58), nursing (OR 0.5, 95% CI: 0.34–0.80), and disabled care (OR 0.6, 95% CI: 0.45–0.90) showed a lower likelihood of hesitation or refusal compared to workers in other fields. Participants from the new federal states were more hesitant than those from the old federal states (OR 1.9, 95% CI: 1.51–2.58). For individuals who had not received the seasonal influenza vaccination in the past 5 years, results showed an increased OR of 5.7 (3.84–7.80) for being hesitant. Surprisingly, individuals who reported frequent contact with COVID-19 patients or clients in particular showed a higher likelihood of not being vaccinated against COVID-19 (OR 1.9, 95% CI: 1.32–2.97).

### 3.3. Reasons for COVID-19 Vaccination

Most individuals chose to be vaccinated to protect their patients, clients, family members, and themselves (Figure 1). Making a positive contribution to infection control and health protection at work was also frequently cited as a reason. Respondents also often agreed with the following reasons: rapid easing of restrictions and more social contacts and travel opportunities. A further 26% of the respondents wanted to be vaccinated due to pre-existing health conditions. Helping to tackle the pandemic and setting a good example were also frequently cited as reasons in the free-text responses.

### 3.4. Reasons for Hesitating or Refusing COVID-19 Vaccination

Participants who wanted to wait before being vaccinated, were undecided, or were inclined to refuse vaccination often stated that they had concerns about short- and long-term consequences or the safety and efficacy of the vaccines. Very few rejected vaccination in general (Figure 2). In the free-text responses, the following reasons were also listed: lack of confidence and criticism of the vaccination policy, perceived social pressure to be vaccinated, and uncertainty with regard to fertility. A total of 65 individuals stated that they had been previously diagnosed with COVID-19. Of these, 43% wanted to be vaccinated or were undecided, while 57% no longer wanted to be inoculated.

### 3.5. Vaccination Willingness in the Workplace and Attitudes toward Mandatory Vaccination

Vaccination willingness in the workplace was described as high by 58% of the respondents, while 31% considered it to be moderate. Just 7% felt it to be low (“don’t know” = 4%). Participating social workers were most likely to describe vaccination willingness as high (71%), while the percentage in geriatric care and nursing stood at 54% and 57% respectively. In answer to the question of what could help improve vaccination willingness in the workplace, respondents agreed with the following points most frequently: detailed information about vaccine safety and how the vaccines work (64%), transparent reporting on possible side effects and long-term consequences (63%), evidence of fewer COVID-19 infections among vaccinated colleagues (60%), and a longer period of observing the vaccination drive to gain a better picture of side effects and long-term consequences (51%). Just 7% advocated a financial incentive for vaccination (no figure). Furthermore, the following reasons (*n* = 334) were cited in free text (listed here by frequency):

- More freedoms for vaccinated individuals and easing of restrictions in the workplace (e.g., fewer tests, no mandatory FFP2/N95 mask);
- Free choice of vaccine;
- Clear rules and a sensible information and vaccination policy;
- Transparent, objective, and balanced reporting;
- Reliable, accessible vaccination options in the workplace (e.g., vaccinations during the working day or vaccinations at work by company doctors or mobile vaccination teams);
- Non-bureaucratic access to vaccination appointments;
- Good information and transparency towards employees;
- Mandatory vaccination for healthcare workers/restrictions for unvaccinated staff;
- Evidence of efficacy and post-vaccination support;
- Role model function for managers and staff;
- No mandatory vaccination.

Around 58% of respondents were against mandatory vaccination. Medical, educational, and therapeutic staff in particular (63% of each group) thought mandatory vaccination was unreasonable.

### 3.6. Information on COVID-19 Vaccination

The majority of respondents (89%) obtained detailed information about the benefits and personal risks of the COVID-19 vaccines. Most obtained information from the websites/information portals of the federal states or the government (74% of cases). Other frequently used sources of information were television, radio, or podcasts (52%), daily newspapers or weekly magazines (print and online; 46%), at the workplace (e.g., notices, information events, newsletters; 29%), or from colleagues (28%). Social media or online video sites (e.g., YouTube), on the other hand, were rarely used to obtain information (18% and 10%, respectively).

## 4. Discussion

Our data showed that a high rate of care workers were already vaccinated. Vaccination willingness stood at a similarly high level among nursing staff and doctors at 84% and 85%, respectively, when vaccinated individuals and those willing to be inoculated were taken together. However, doctors were under-represented in this sample. The vaccination rate and willingness to be inoculated against COVID-19 were high overall across all professional groups and fields of work (over 80%). Although the percentage of individuals who refused to be vaccinated varied by age and profession, it was low among all subgroups at a maximum of 14%. Furthermore, it is possible that among the unvaccinated, willingness to be vaccinated tends to decline, suggesting that many of those willing to be vaccinated are already immunized. As expected, vaccination willingness was higher among older workers because the health risk posed by a SARS-CoV-2 infection is considerably greater for them. Our results are also comparable to those from the general population with regard to vaccination status, willingness, and vaccine preference. Since May 2020, COVID-19 Snapshot Monitoring (COSMO) has been conducted at regular intervals among the population in Germany. In addition to other pandemic-relevant topics, vaccination readiness is repeatedly surveyed. The authors have found that vaccination readiness in the population has risen continuously since December 2020 (average 50%) and currently stands at 78% (June 2021; consisting of those already vaccinated and those willing to be vaccinated). The BioNTech/Pfizer vaccine was most preferred (51%), while 1.4% preferred the AstraZeneca vaccine. About a quarter had no preference for a certain vaccine [21].

The willingness to be inoculated against COVID-19 was higher among individuals who had received a seasonal flu vaccination than among those who had not been inoculated against flu. This was also to be expected. Of those who had been vaccinated against influenza at least once in the past five years, the majority had also been inoculated against SARS-CoV-2 or intended to be immunized. However, at 77%, the willingness to receive a COVID-19 vaccination was also high among individuals who had not been inoculated against influenza. Our data confirm the observation by Spinewine and colleagues that individuals who receive influenza vaccines also show positive attitudes toward COVID-19 vaccines [22]. Using the 5C theoretical model to determine psychological antecedents for vaccination, Kwok et al. showed that influenza vaccination was associated with stronger vaccination confidence, collective responsibility, and weaker risk perception (complacency), perceived barriers (constraints), and benefit-risk trade-offs (calculation). Similarly, stronger vaccination confidence, collective responsibility, and weaker complacency were predictive of COVID-19 vaccination [19].

Vaccinations are a crucial means of controlling infection in the workplace to protect workers’ health. Since HCWs in particular are on the front line in the fight against COVID-19, they are at a high risk of infecting themselves or other vulnerable groups. According to a meta-analysis, nursing staff were the most frequently affected group of workers at 48% [23]. However, surveys showed that the vaccination willingness among nursing staff in particular was low [11,13,15,19]. At the beginning of the vaccination campaign, vaccination willingness rates stood between 50% and 70% depending on the professional group. Just a few weeks after the vaccination campaign began, however, a constant rise in vaccination willingness was observed in all professional groups [12,14,24]. There are various reasons for the increase. Firstly, observation of the efficacy of vaccination at population level [25] or among healthcare workers [26,27] may have increased vaccination willingness. Secondly, individuals’ confidence in the safety and efficacy of the vaccines may have grown as more and more people in their immediate environment were vaccinated against COVID-19 [28]. Social desirability may have increased vaccination willingness, especially for inoculations at healthcare facilities. Alongside infection protection, our data show that the prospect of further easing restrictions and more opportunities for social contact are important reasons for vaccination. This is tied to the high expectations people have for vaccinations. Health protection remains the primary objective of the vaccination campaign: restrictions can only be eased consistently when the vaccination rate is high enough to end the pandemic.

In our survey, almost 60% oppose mandatory vaccination. This is also consistent with the results of regular monitoring surveys of the population in Germany. Only 1/3 of respondents were in favor of mandatory vaccination for HCWs. It is suspected that mandatory vaccination may have psychological side effects. If support for mandatory vaccination is low in the population, an imposed requirement to vaccinate may lead to reactance, which in turn may negatively affect compliance with other protective measures in the population [29]. In Germany, there is no general obligation to vaccinate against COVID-19, not even for professions with a high risk of infection. However, the federal government advocates for a strong vaccination recommendation to ensure protection at the individual and community level [3]. Nevertheless, it is possible that in some cases societal expectations and pressure from employers may lead to de facto mandatory vaccination.

Although it was a small number (*n* = 43), surprisingly, those who had regular contact with COVID-19 patients were more hesitant to be vaccinated. This could be due to the assumption that they already have natural immunity without knowing that vaccination boosts the immune system after infection and is therefore recommended.

The concerns cited by the respondents with regard to the COVID-19 vaccines were largely shared with participants in other studies [16,17,18,24]. To counteract these concerns, it may be helpful to highlight what is known so far about the adverse effects of COVID-19, so that people can weigh the risks and benefits of vaccination.

Online surveys usually have limitations. Generalization of the results should be made with caution, as we used an opportunity sample with self-selection of participants. Besides, the population of heterogeneous occupational groups cannot be fully modeled. This may have resulted in selection bias. The response rate could not be calculated because it was not possible to establish a clearly defined number of potential respondents in advance. It is also difficult to assess how representative the findings are because the socio-demographic data cannot be validated. Another limitation of web-based surveys can result from a high proportion of interview dropouts. In the current survey, the completion rate was moderate at 66.7%. We cannot rule out the possibility that individual participants completed the questionnaire more than once.

Furthermore, it is difficult to judge whether there was a difference in willingness among vaccination sceptics or vaccination supporters to participate in the survey. However, we can see no evidence to indicate that more vaccination supporters than vaccination sceptics took part.

It is also common for participants in online surveys to not provide their socio-demographic details or to terminate their participation prematurely, despite the anonymous survey design. However, we believe that that happened rarely with this particular survey. Furthermore, we cannot rule out the possibility that an individual took part more than once. Other limitations exist in that we collected no data on professional experience, occupational stress in connection with the COVID-19 pandemic, or vaccination side effects.

## 5. Conclusions

This survey took part at the height of the third wave in Germany, during which the vaccination campaign picked up pace. However, the campaign was thwarted by the report of serious side effects following immunization with the AstraZeneca vaccine. Nevertheless, these data show that health and welfare workers have weighed the risks of vaccination against the possible consequences of COVID-19 and that they demonstrate great willingness to be vaccinated despite the reported side effects. Our data suggest that a vaccination rate of over 80% has already been partially achieved or will soon be achieved among health and welfare workers. In all professional groups, the vaccination rate and vaccination willingness are well above the vaccination rate of 60% to 70% cited by the World Health Organization (WHO) as being necessary to achieve herd immunity [30]. Furthermore, COVID-19 immunization appears to enjoy a higher level of acceptance than influenza vaccinations because the focus is not just on health protection, but also on actively helping to tackle the pandemic. Nonetheless, young people and women remain hesitant about the new vaccines, primarily citing concerns about the long-term effects, safety, and efficacy of the vaccines being used. Workers in these professions will weigh the risks for the patients, residents, or clients they assist as well as their own convictions and personal concerns when they make decisions about COVID-19 vaccinations. These concerns and fears should therefore be taken seriously and addressed by means of transparent information and communication campaigns. Even if the willingness to vaccinate and the vaccination rate in some professions and settings is already above 80%, the subjective perception of vaccination readiness within the company is still perceived by many as moderate or low. Here, practical barriers need to be removed so that the effort to get vaccinated is low, e.g., through uncomplicated vaccination appointments, vaccination at the workplace, or free choice of vaccine. It is likely that as the vaccination campaign progresses, barriers to vaccination will increase among those who are still unvaccinated. Here, targeted information that is understandable to laypersons should be provided so that an individual risk assessment can be made. In our survey, the majority of respondents listed protection of others as a motivation for vaccination. The role of protecting others should be used more prominently in communicating the benefits of vaccination. The majority of respondents cite traditional media as their main source of information related to COVID-19 vaccination. These media should continue to be used to provide evidence-based information to target groups (e.g., younger people) about the safety, risks, and benefits of vaccination.

## Figures and Tables

**Table 1:** Description of the study population.

	Frequency	Percent ^1^
	*n*	%
**Gender**		
Female	2371	70.4
Male/Diverse	997	29.6
**Age (years)**		
<35	479	14.2
35–55	1791	52.9
>55	1114	32.9
**Professional group**		
Nursing staff	908	27.1
Medical staff	148	4.4
Therapeutic staff	353	10.5
Administrative staff	967	28.9
Educational/support staff	541	16.2
Body-related service staff	72	2.2
Other professions	359	10.7
**Field of work**		
Geriatric care	1018	30.1
Nursing	352	10.4
Work with disabled people	453	13.4
Social work	381	11.3
Other areas	1173	34.7
**Federal states**		
Western states	2897	85.9
Eastern states	476	14.1

Note: ^1^ Valid percentages. Missing values: gender (*n* = 33, 1%), age (*n* = 17, 0.5%), professional group (*n* = 53, 1.6%), working field (*n* = 24, 0.7%), federal states (*n* = 28, 0.8%).

**Table 2:** Vaccination status and willingness of unvaccinated individuals for COVID-19 vaccination.

			Vaccination Status and Willingness				*p*-Value
			Vaccinated	Accepting	Hesitating/Undecided	Refusing	
			*n* = 2108	*n* = 761	*n* = 224	*n* = 308	
Gender	Female	*n*	1490	505	169	207	<0.05
		%	62.8	21.3	7.1	8.7	
	Male/Diverse	*n*	601	247	53	96	
		%	60.3	24.8	5.3	9.6	
Age (years)	<35	*n*	262	99	51	67	<0.001
		%	54.7	20.7	10.6	14.0	
	35–55	*n*	1124	379	113	175	
		%	62.8	21.2	6.3	9.8	
	>55	*n*	713	280	59	62	
		%	64.0	25.1	5.3	5.6	
Professional group	Nursing staff	*n*	680	78	60	90	<0.001 *
		%	74.9	8.6	6.6	9.9	
	Medical staff	*n*	98	27	6	17	
		%	66.2	18.2	4.1	11.5	
	Therapeutic staff	*n*	225	64	24	40	
		%	63.7	18.1	6.8	11.3	
	Administrative staff	*n*	617	230	51	69	
		%	63.8	23.8	5.3	7.1	
	Educational/support staff	*n*	283	185	45	28	
		%	52.3	34.2	8.3	5.2	
	Body-related service staff	*n*	9	52	8	3	
		%	12.5	72.2	11.1	4.2	
	Other professions	*n*	172	108	28	51	
		%	47.9	30.1	7.8	14.2	
Field of work	Geriatric care	*n*	823	68	55	72	<0.001
		%	80.8	6.7	5.4	7.1	
	Nursing	*n*	262	39	19	32	
		%	74.4	11.1	5.4	9.1	
	Work with disabled people	*n*	273	110	26	44	
		%	60.3	24.3	5.7	9.7	
	Social work	*n*	151	171	31	28	
		%	39.6	44.9	8.1	7.3	
	Other fields of work	*n*	593	366	90	124	
		%	50.6	31.2	7.7	10.6	
Federal states	Western states	*n*	1837	640	186	234	<0.001
		%	63.4	22.1	6.4	8.1	
	Eastern states	*n*	261	111	37	67	
		%	54.8	23.3	7.8	14.1	
Influenza vaccination (<5 years)	Every year	*n*	544	187	27	13	<0.001
		%	70.6	24.3	3.5	1.7	
	Once or irregularly	*n*	578	186	40	35	
		%	68.9	22.2	4.8	4.2	
	None	*n*	983	388	157	257	
		%	55.1	21.7	8.8	14.4	
Contact to patients with COVID-19 (<4 weeks)	Never/rarely	*n*	1972	743	210	272	<0.001
		%	61.7	23.2	6.6	8.5	
	Often/always	*n*	126	16	12	31	
		%	68.1	8.6	6.5	16.8	

Note: Line percentages are indicated; * Fisher’s exact test.

**Table 3:** Factors associated with hesitancy or refusal to be vaccinated against COVID-19.

			Vaccinated/ Accepting (*n* = 2869)	Hesitating/Refusing (*n* = 532)	OR	95% CI	*p*-Value
Gender	Female	*n*	1995	376	0.9	0.77–1.22	n.s.
		%	84.1	15.9			
	Male/Diverse	*n*	848	149	1	-	
		%	85.1	14.9			
Age (years)	<35	*n*	361	118	1.9	1.40–2.59	<0.001
		%	75.4	24.6			
	35–55	*n*	1503	288	1.3	1.04–1.69	
		%	83.9	16.1			
	>55	*n*	993	121	1	-	
		%	89.1	10.9			
Professional group	Nursing staff	*n*	758	150	1.1	0.73–1.63	<0.01
		%	83.5	16.5			
	Medical staff	*n*	125	23	0.8	0.48–1.49	
		%	84.5	15.5			
	Therapeutic staff	*n*	289	64	0.7	0.49–1.1	
		%	81.9	18.1			
	Administrative staff	*n*	847	120	0.6	0.47–0.96	
		%	87.6	12.4			
	Educational/support staff	*n*	468	73	0.5	0.34–0.78	
		%	86.5	13.5			
	Body-related service staff	*n*	61	11	0.5	0.25–1.07	
		%	84.7	15.3			
	Other professions	*n*	280	79	1	-	
		%	78.0	22.0			
Field of work	Geriatric care	*n*	891	127	0.4	0.30–0.58	<0.001
		%	87.5	12.5			
	Nursing	*n*	301	51	0.5	0.34–0.80	
		%	85.5	14.5			
	Work with disabled people	*n*	383	70	0.6	0.45–0.90	
		%	84.5	15.5			
	Social work	*n*	322	59	0.8	0.60–1.27	
		%	84.5	15.5			
	Other fields of work	*n*	959	214	1	-	
		%	81.8	18.2			
Federal states	Eastern states	*n*	372	104	1.9	1.51–2.58	<0.001
		%	78.2	21.8			
	Western states	*n*	2477	420	1	-	
		%	85.5	14.5			
Influenza vaccination (<5 years)	Every year	*n*	731	40	1	-	<0.001
		%	94.8	5.2			
	Once or irregularly	*n*	764	75	1.7	1.17–2.67	
		%	91.1	8.9			
	None	*n*	1371	414	5.4	3.84–7.80	
		%	76.8	23.2			
Contact to patients with COVID-19 (<4 weeks)	Never/rarely	*n*	2715	482	1	-	<0.01
		%	84.9	15.1			
	Often/always	*n*	142	43	1.9	1.32–2.97	
		%	76.8	23.2			

## Data Availability

Data are available from a.kozak@uke.de.
